# A Comparison of mRNA Sequencing with Random Primed and 3′-Directed Libraries

**DOI:** 10.1038/s41598-017-14892-x

**Published:** 2017-11-07

**Authors:** Yuguang Xiong, Magali Soumillon, Jie Wu, Jens Hansen, Bin Hu, Johan G. C. van Hasselt, Gomathi Jayaraman, Ryan Lim, Mehdi Bouhaddou, Loren Ornelas, Jim Bochicchio, Lindsay Lenaeus, Jennifer Stocksdale, Jaehee Shim, Emilda Gomez, Dhruv Sareen, Clive Svendsen, Leslie M. Thompson, Milind Mahajan, Ravi Iyengar, Eric A. Sobie, Evren U. Azeloglu, Marc R. Birtwistle

**Affiliations:** 1 Department of Pharmacological Sciences and DToxS LINCS Center, Icahn School of Medicine at Mount Sinai, New York, NY USA; 2 Broad Institute of MIT and Harvard, Cambridge, MA USA; 3 Department of Biological Chemistry, University of California, Irvine, CA USA; 4 UCI MIND, University of California, Irvine, CA USA; 5 Board of Governors-Regenerative Medicine Institute, Cedars-Sinai Medical Center, Los Angeles, CA USA; 6 iPSC Core, The David and Janet Polak Foundation Stem Cell Core Laboratory, Los Angeles, CA USA; 7 Department of Biomedical Sciences, Cedars-Sinai Medical Center, Los Angeles, CA USA; 8 Department of Psychiatry and Human Behavior, Neurobiology and Behavior, University of California, Irvine, CA USA; 9 Department of Genetics, Icahn School of Medicine at Mount Sinai, New York, NY USA; 10 Present Address: Berkeley Lights, Inc. 5858 Horton St., Emeryville, CA 94608 USA; 11 Present Address: Department of Chemical and Biomolecular Engineering, Clemson University, Clemson, SC USA

## Abstract

Creating a cDNA library for deep mRNA sequencing (mRNAseq) is generally done by random priming, creating multiple sequencing fragments along each transcript. A 3′-end-focused library approach cannot detect differential splicing, but has potentially higher throughput at a lower cost, along with the ability to improve quantification by using transcript molecule counting with unique molecular identifiers (UMI) that correct PCR bias. Here, we compare an implementation of such a 3′-digital gene expression (3′-DGE) approach with “conventional” random primed mRNAseq. Given our particular datasets on cultured human cardiomyocyte cell lines, we find that, while conventional mRNAseq detects ~15% more genes and needs ~500,000 fewer reads per sample for equivalent statistical power, the resulting differentially expressed genes, biological conclusions, and gene signatures are highly concordant between two techniques. We also find good quantitative agreement at the level of individual genes between two techniques for both read counts and fold changes between given conditions. We conclude that, for high-throughput applications, the potential cost savings associated with 3′-DGE approach are likely a reasonable tradeoff for modest reduction in sensitivity and inability to observe alternative splicing, and should enable many larger scale studies focusing on not only differential expression analysis, but also quantitative transcriptome profiling.

## Introduction

Massively parallel sequencing of mRNA, or mRNAseq, was first introduced in 2008^1–3^, and since has rapidly become the preferred method for whole transcriptome measurements^4–11^, culminating recently with the announcement by Illumina of the discontinuation of the Human Expression Array BeadChip (HumanHT-12 v4 as of 9 Dec 2016). The procedure consists of two basic steps. First is library preparation, which consists of converting mRNA isolated from samples into cDNA that is compatible with the deep sequencing platform. Next is the sequencing itself, which often consists of paired-end protocols on the Illumina HiSeq platform to generate millions of reads per sample. The resulting quantification of transcript levels comes from counting the reads aligned to each transcript, followed with normalizing by transcript length (since # of reads depends on transcript length due to random priming), and the total number of reads (sequencing depth)^5,11^. Analysis of alternative splicing is improved with paired-end sequencing compared to single-end sequencing. This is because paired-end sequencing puts strong constraints on the distance between reads with respect to the reference transcriptome, giving better discrimination amongst known splice isoforms^2,5^.

Although the data resulting from mRNAseq is often considered superior to that obtained by the former transcriptome measurement standard, the microarray, the cost per sample still precludes widespread high-throughput application. A primary component of this cost is the library preparation. Thus, reduction in library preparation cost is expected to pay large returns for ability to increase mRNAseq throughput.

Another current issue with mRNAseq data is the biases introduced by PCR during library preparation. Because every sequence has a potentially different propensity to be amplified during PCR, the resulting quantitative representation of transcripts in the sequencing library is non-linearly distorted from the original abundance in ways that are difficult to predict. One way that has been shown to correct for this bias is to tag every transcript molecule with a unique random nucleotide sequence prior to amplification^12–14^. Such sequences are termed unique molecular identifiers (UMIs). UMIs allow removal of PCR bias by counting only the reads for a gene that have different UMI sequences, and ignoring those that have the same UMI sequence, since they came from the same original transcript molecule and thus are PCR duplicates. For this reason, such quantification approaches are also often referred to as transcript counting or digital gene expression (DGE). Prior work has shown that UMI counting is the only type of amplification correction that improves statistical properties of the data, including variance, power and false discovery rate^15^. These are important reasons why UMI-based DGE is a method of choice for single cell mRNA sequencing^16^, where amplification after sample pooling (as is done in this manuscript) seems to improve statistical properties^15^.

Here, we demonstrate the use of a library preparation method that takes advantage of 3′-end creation of cDNA (poly-T priming) and incorporates UMI-based quantification, but in our particular case, significantly reduces the cost of library preparation and thus the per sample cost. Because it is not yet clear to what extent the results from this 3′-DGE library preparation method overlap with the current gold-standard of random primed conventional mRNA sequencing, we performed a comprehensive comparison of data obtained by both methods, from the same RNA samples. For our samples, we find that 3′-DGE has only about ~15% lower sensitivity than conventional random primed mRNAseq, good quantitative agreement, and high overlap in ranked lists of differentially expressed genes. We conclude that the 3′-DGE approach used here is likely to be a viable alternative to conventional random-primed mRNAseq for high-throughput applications, particularly when looking for differentially expressed genes between treatment conditions, as is a common goal for transcriptomic studies. This is also relevant for more simple expression profiling which is becoming more commonplace in single-cell mRNAseq^17–20^ or tissue-level examinations^21–23^. Such single-cell mRNAseq applications of 3′-DGE are also relevant for distinguishing between cell types within a tissue or population, which based on the results of this study, we expect to be broadly covered. We also find that 3′-DGE can identify most, if not all, relevant mRNA species as compared to conventional random primed mRNAseq, so long as low copy number transcripts are able to be reverse transcribed.

## Results and Discussion

### 3′-Digital Gene Expression Versus Conventional mRNA Sequencing

**Figure 1:**
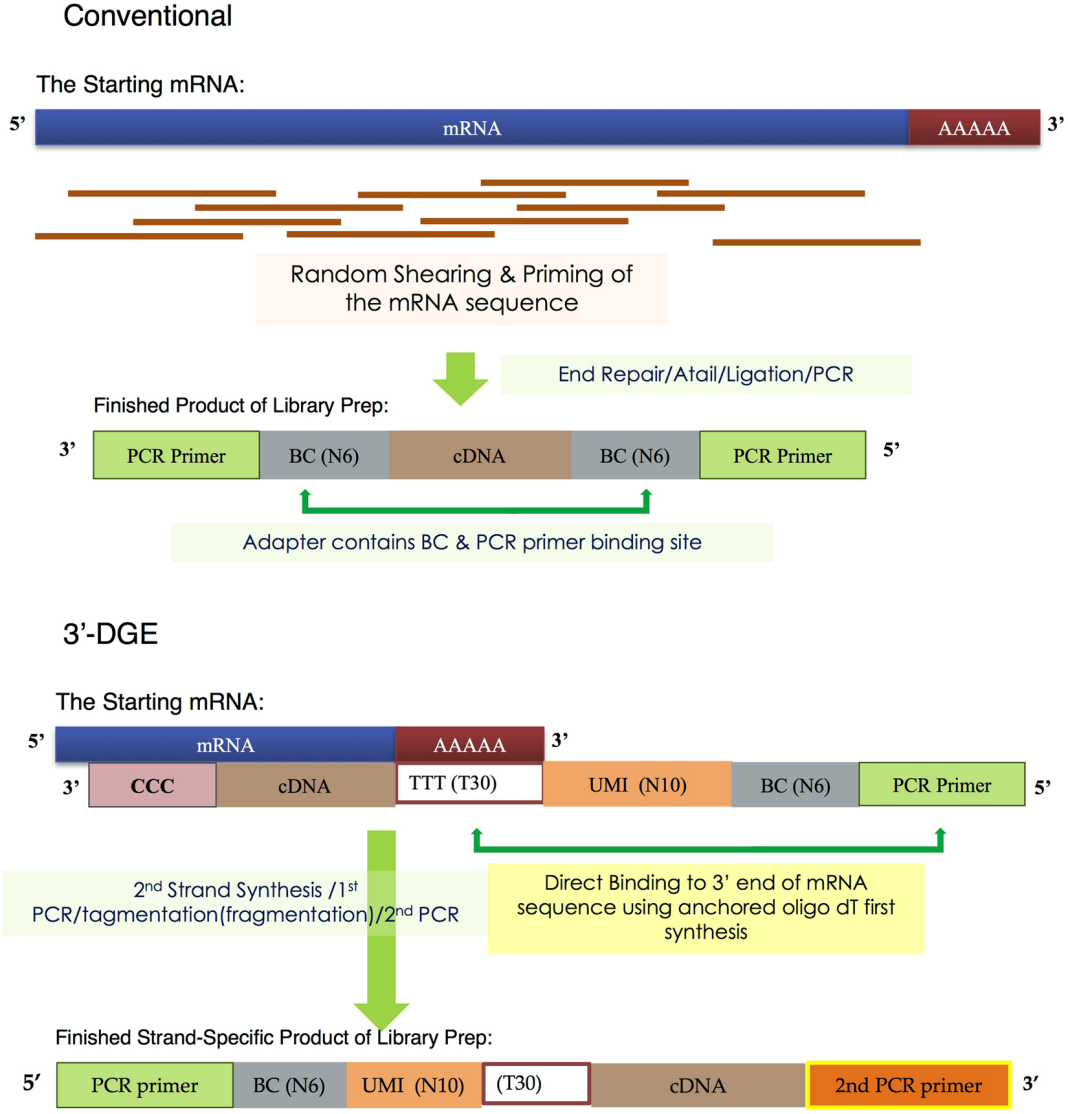
Schematic of Library Construction Differences between Conventional and 3′-DGE mRNA Sequencing. BC: Barcode; UMI: Unique Molecular Identifier.

Both conventional and 3′-DGE mRNAseq consist of (i) library preparation, (ii) sequencing, (iii) alignment to a reference genome and (iv) quantification; but there are differences between the two approaches in each of these four main steps (Fig. 1 illustrates some of these). For conventional library preparation, following isolation of mRNA from total RNA (in our case with oligo dT beads—see Methods), cDNA is typically synthesized via random priming of thermally-sheared mRNA. In 3′-DGE, cDNA is generated via 3′-directed oligo dT priming, during which unique molecular identifiers (UMI) are incorporated (for quantification purposes—see below), and strand-specificity is preserved. For conventional sequencing, single-end or paired-end constant read length is used (we used 100 bp single-end in this paper). In 3′-DGE, paired-end is required. At least 16 bp are needed on the first read to capture UMI and sample barcode data. We used 46 bp on the second read to acquire transcript-specific sequence data. Focus on the 3′ end of the transcript coupled with lack of transcript-specific information on both reads precludes identification of alternative splicing for 3′-DGE. For conventional alignment, a the comprehensive reference genomeis used since sequenced fragments are randomly distributed. In 3′-DGE, an mRNA RefSeq transcriptome is used (see Methods). Because of this, there are slight differences in the gene lists obtained from each alignment (Table S1), but a large majority of protein-coding genes (22,811) are shared between the approaches. For conventional quantification, the total number of uniquely aligned reads to a particular gene is used, termed read counts. Read counts are often divided by the average transcript length and read depth (units of **r**eads **p**er **k**ilobase of transcript length per **m**illion mapped reads—RPKM). In 3′-DGE, the total number of uniquely aligned reads to particular genes is available, but is only an intermediate to the final quantification by UMI counts for each gene. This UMI count metric corrects for PCR bias by removing reads that align to the same genomic region and share the same UMI sequence (this strongly suggests they arose from the same original transcript molecule).

To gain a thorough understanding of the practical differences between data obtained by 3′-DGE and conventional mRNAseq, we isolated RNA from 16 PromoCell primary cardiomyocyte –like cultures treated with either DMSO vehicle control (eight biological replicates), sorafenib (four biological replicates) or sunitinib (four biological replicates) for 48 hours. These experiments were part of a larger signature generation project in our DToxS LINCS center focused on cellular signatures for cardiotoxicity of kinase inhibitors (www.dtoxs.org and www.lincsproject.org). We expected, based on our prior data, that sorafenib would induce large changes in gene expression, whereas sunitinib would induce negligible changes in gene expression, providing positive and negative control test cases for differential expression analysis. The 16 RNA samples were analyzed for quality, and then split and sent to either the conventional or 3′-DGE mRNAseq pipeline for library preparation, sequencing, alignment and quantification (see Methods and Fig. S1). The average read depth for conventional was 5.9 × 10^6^ reads/sample, and for 3′-DGE was 3.8 × 10^6^ reads/sample. Read depth was also consistent across samples (Fig. S2), and overall read count distribution was similar for the two techniques (Fig. S3).

### Fidelity of Sequence Alignments for 3′-DGE

**Figure 2:**
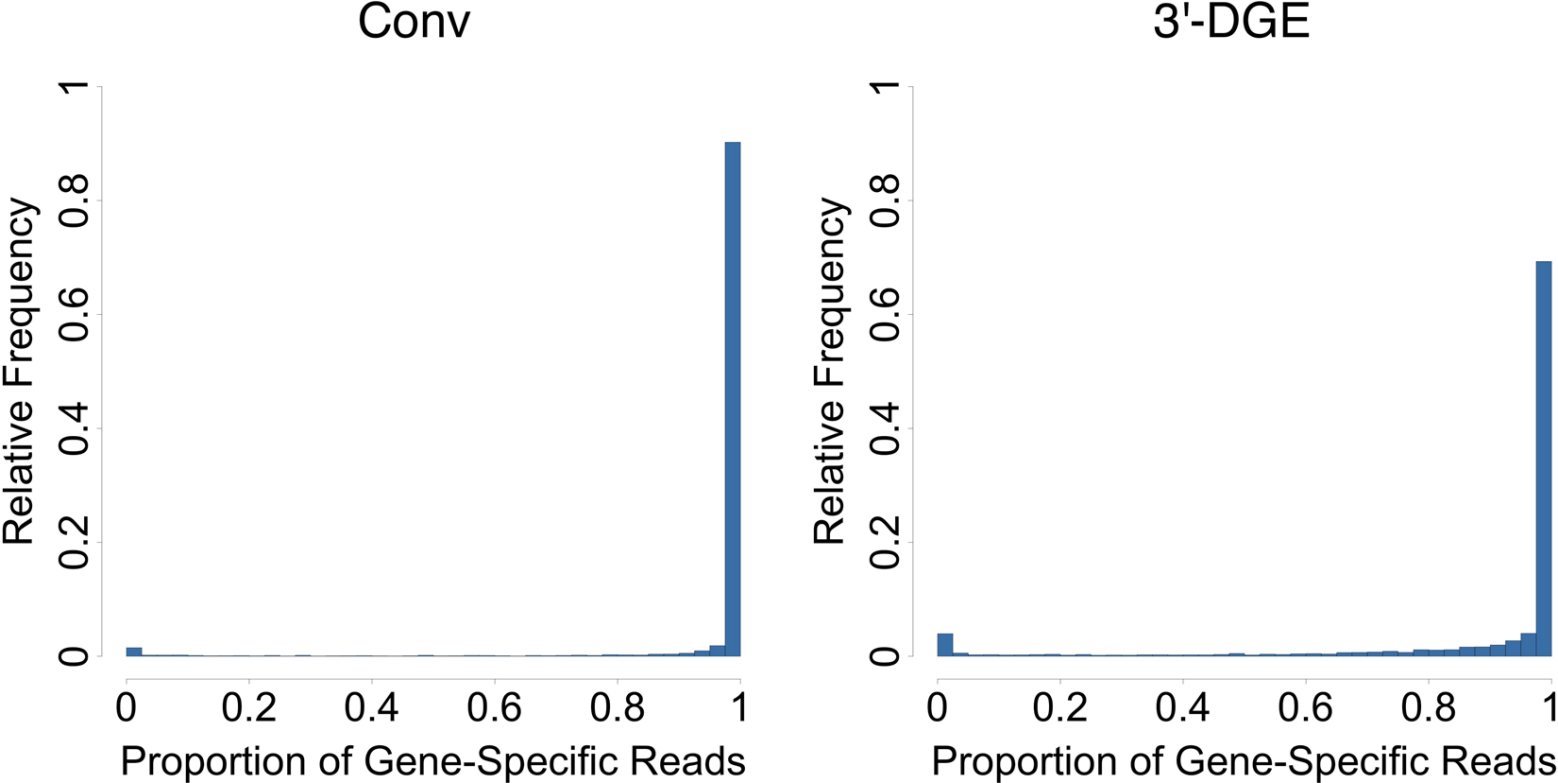
Fidelity of Sequence Alignments in Conventional (Conv) and 3′-end Digital Gene Expression (3′-DGE) Method. For each of the ~14,000 genes with greater than four counts (summed across all 16 samples) measured by conventional method and 3′-DGE method, the proportion of reads aligned to only that one gene was calculated. This proportion is close to 1 for most genes, indicating reliable quantification.

Because sequence information in 3′-DGE comes from a restricted region of the exome that may have reduced sequence entropy amongst genes, we first investigated the fidelity of sequence alignments. For each gene with at least four read counts (summed across all samples), we quantified the proportion of reads that align only to that gene, and looked at the frequency distribution of this proportion across genes (Fig. 2). The distribution is highly skewed towards proportions >0.95, indicating most genes are quantified by reads that align only to that single gene. This proportion is less than 0.10 for a small number of genes (738 out of 14,574; gene names are in Table S2). Thus, these 738 genes are not able to be reliably quantified without further assumptions and considerations. The reads associated with these unquantifiable genes account for most such degenerately-aligned reads (>50%). We conclude from these data that despite the fact that reads are 3′-end-focused, a large and sufficient majority can be reliably mapped to individual genes with high fidelity. This feature may be facilitated by the strand-specificity of the 3′-DGE library preparation method. From this point forward we only consider read counts that are reliably aligned to a single gene.

### Sensitivity to Detect Gene Expression as a Function of Read Depth

**Figure 3:**
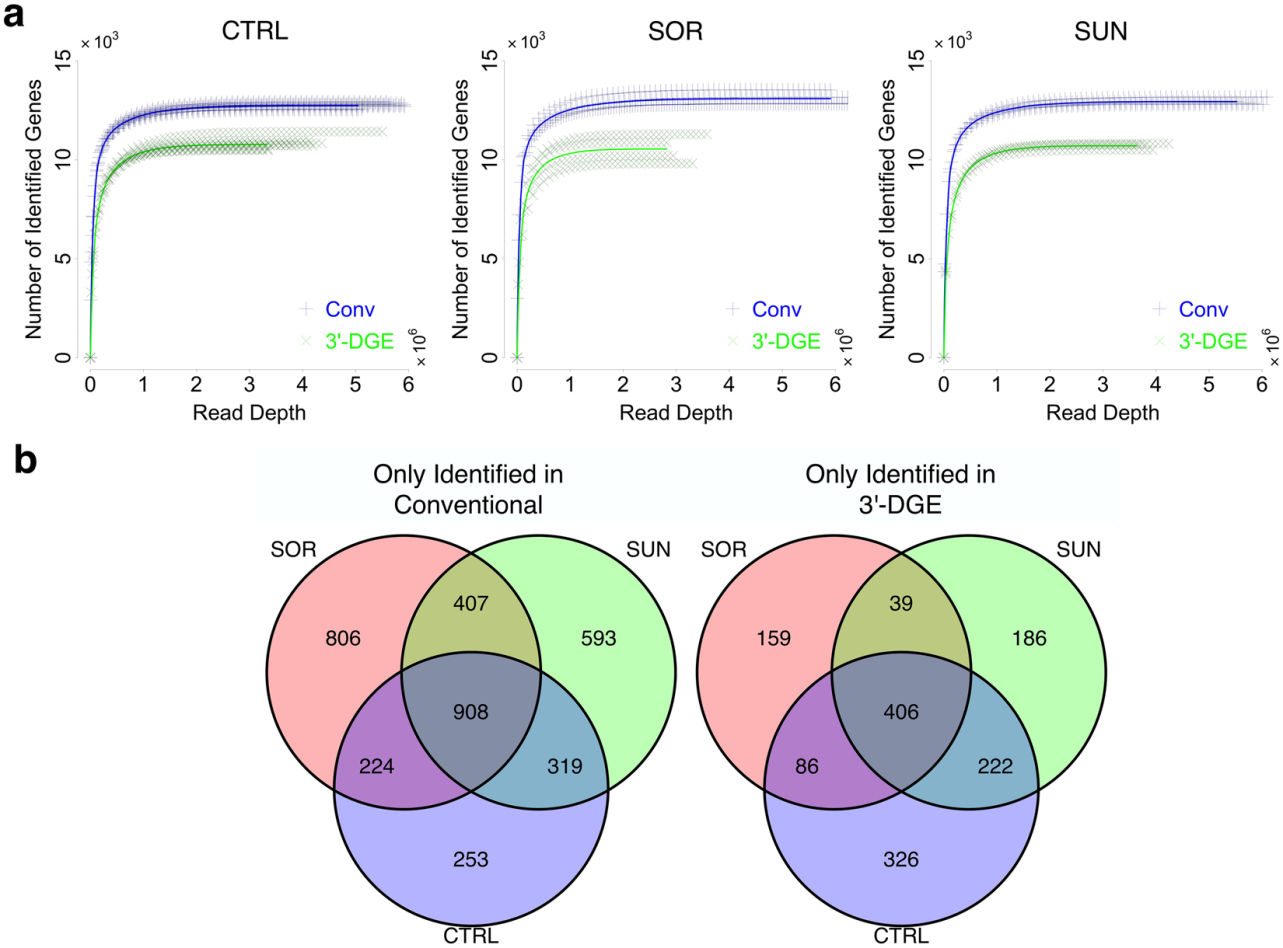
Sensitivity of Gene Detection by Conventional (Conv) and 3′-end Digital Gene Expression (3′-DGE) mRNA Sequencing Methods. (**a**) Gene-wise reads are removed from every sample in a probability proportional to the abundance of the gene in a sample, to generate a set of the number of identified genes over a range of simulated read depths. The curves for individual replicate samples are shown with the thinner points, showing in general low variability. The average is shown with the solid line. (**b**) Each replicate from both mRNAseq technologies were downsampled via read removal to a common read depth (2.8 million reads per sample), and the differences in identified genes were analyzed. Most genes identified in conventional but not 3′-DGE were shared across treatment conditions, and likewise for those identified by 3′-DGE but not conventional. Specific gene names are listed in Tables S3,S4.

We wanted to determine the sensitivity of the two techniques to detect expression of the 22,811 genes shared between the reference sequence databases. To do this we employed a read removal approach, where each sample’s dataset was progressively downsized (Fig. 3a and Figs. S4,S5). Read counts were removed from each gene with probability proportional to that gene’s overall representation in the dataset, and expression of a gene was considered detected with four or greater reads (see Methods). We verified that this stochastic nature of read removal introduced negligible variability into our results (Fig. S6).

In all treatment cases, we observed a hyperbolic dependency between read depth and number of detected genes. We parameterized these relationships with a Michaelis-Menten model to quantify differences between the techniques (Table 1). We found that the estimated parameter values were consistent across samples for each measurement type. The *V*_*max*_ parameter indicates that on average conventional mRNAseq detects approximately 15% (~2000) more genes. The genes that were differentially detected by conventional were very consistent across treatments, and were greater in number than those differentially detected by 3′-DGE as expected (Fig. 3b). The overall fraction of genes identified by only conventional or 3′-DGE was small, indicating good concordance between the techniques (>10^4^ vs. <10^3^). The *K*_*m*_ parameter indicates that on average conventional mRNAseq is slightly more sensitive; however, at read depths even far below typical levels (10^6^/sample), both techniques detect ~95% of the maximum possible.

**Table 1:** The Best-Fit Parameter Values of a Michaelis-Menten Model to the Average Curves in Fig. 3a. The function *drm* in the R package *drc*, using the fit function *mm*, was used.

Technology	Treatment	Km (reads)	Vmax (genes)
Conv	CTRL	49,719	12,905
Conv	SOR	45,441	13,202
Conv	SUN	50,902	13,099
3′-DGE	CTRL	75,277	11,117
3′-DGE	SOR	60,979	10,884
3′-DGE	SUN	70,673	11,006

Given that we cannot (or did not) observe a particular set of genes for a given technique, what is the loss of power to detect enrichment of gene ontology terms? We answered this question both on the level of what is possible to observe with conventional or 3′-DGE (Table S7), or what we did observe in our particular dataset (Fig. S9). We find there are very few biological processes that go from above to below 80% power for detection. Olfactory and other sensory processes, some histone regulatory processes and some mitochondrial processes are harder to identify with 3′-DGE, with other processes showing less loss of power, which to some extent are addressable with increased sequencing depth.

We conclude that conventional mRNAseq detects the expression of approximately 15% more genes (~13,000 vs. ~11,000), but both techniques saturate in terms of detecting new genes at greater than 2-3 million reads/sample. We stress however that these results are specific to our particular experimental system (human primary cardiomyocyte cell lines), the reference library and technical apparatus, which can alter the sequencing depth that is needed to reach saturation of detection. While prior work has suggested detection saturates at much higher depth that includes non-coding sequences which we do not consider here; coding sequences tend to saturate much more quickly^24^. It should be noted here that the 3′-DGE method was designed to be compatible with very little total RNA extract (e.g. single cell RNAseq), and also undergoes fewer PCR cycles (10 vs. 15) in our protocols, so this may contribute to the differences observed here. In addition, detection is strongly distinct from statistical power, so 2-3 million reads/sample may not be sufficient to detect differential expression of lowly expressed genes between two conditions, despite being detected. Differential expression and power also depend strongly on the number of biological replicates, the number of which we use (eight for controls and four for samples), is close to ideal as studied previously^25^.

### Quantitative Comparison Between the Techniques

**Figure 4:**
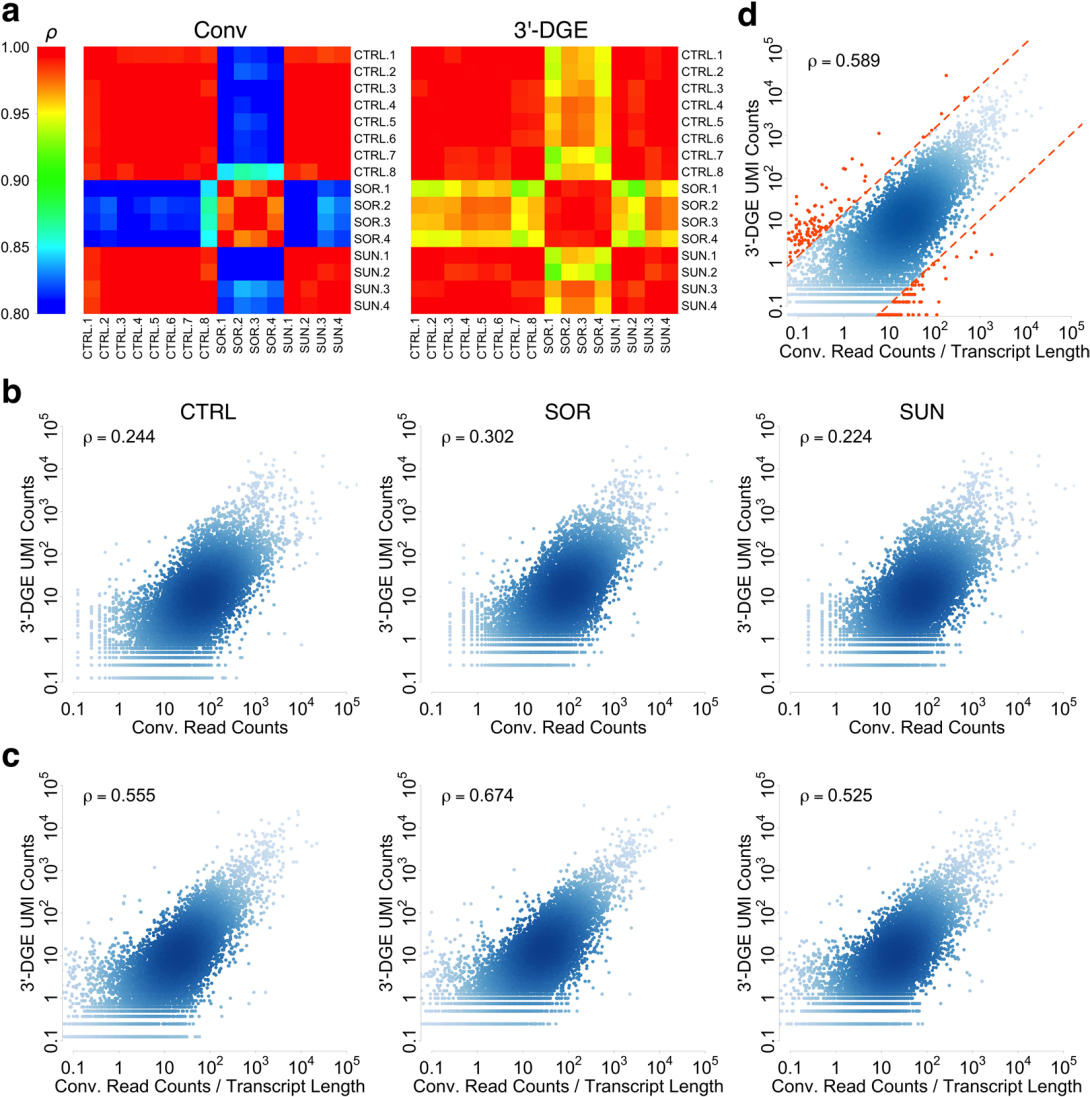
Quantitative Comparison between Conventional (Conv) and 3′-end Digital Gene Expression (3′-DGE) mRNA Sequencing Methods. (**a**) Correlations of the replicate samples from the Conv read counts and 3′-DGE read counts show that the replicate samples obtained by the same method correlate well with each other under each condition. Control (CTRL), Sorafenib (SOR), Sunitinib (SUN). Pearson correlation is used. (**b**,**c**) Quantitative gene-wise comparison between Conv read counts and 3′-DGE UMI counts. Datasets are downsampled to a common read depth of 2.8 million reads, and then gene-by-gene comparisons are made via scatter plots. To generate a reduced UMI count dataset, upon removal of a read count, UMI counts were removed with probability proportional to the ratio between UMI counts and read counts for that gene (accounting for PCR bias). Density of points in scatter plots is indicated by depth of color. Inset text box shows Pearson correlation. In all plots, data are scaled so units are comparable. (**b**) Scatterplots of UMI counts for 3′-DGE versus read counts for conventional, without normalization by average transcript length. There is a general trend of agreement but correlation is low for quantitative agreement. (**c**) Scatterplots of UMI counts for 3′-DGE versus transcript length-normalized read counts for conventional. Quantitative agreement is significantly improved upon this normalization. (**d**) Potential biases of 3′-DGE or Conv techniques. We averaged data from all 16 read depth-normalized samples and defined lines that flank the typical variance in the data to identify genes that have evidence of bias in quantification. Genes falling outside of this range are reported in Tables S5,S6.

We next wanted to do a quantitative comparison of gene-by-gene expression for matched samples and treatment conditions. The first step in doing so is to normalize read depth amongst all the samples. To do that, we used the read removal process as above to downsample each dataset to the lowest common read depth, which was ~2.8 million reads per sample. Correlation amongst replicates within the same technique was very high (Fig. 4a), but the resulting x-y scatterplots of conventional read counts vs. DGE read counts yielded poor correlation (Fig. 4b). We reasoned that this poor correlation could be due to the fact that conventional read counts depend strongly on transcript length, whereas in principle, those from 3′-DGE do not. When we normalized conventional read counts by transcript length, the correlation improved significantly (Fig. 4c). We observed similar agreement on the level of individual sample-to-sample correlations (Fig. S7). We conclude that the two techniques show reasonable quantitative agreement with one another.

We also used such scatter plot analysis to yield insight into potential biases in detection for each technique (Fig. 4d). We first took the average read count values for each gene across all 16 samples, with the thinking that strong biases should largely be sample independent. As a simple and conservative approach, we drew lines parallel to x = y that flank the typical variance in the data, and identified points falling outside of this range (Fig. 4d). We identified a small relative number of such genes—165 for 3′-DGE and 98 for conventional (Tables S5,S6). These genes should be interpreted with caution when appearing as differentially expressed in downstream analyses.

### Differential Expression Analysis

**Figure 5:**
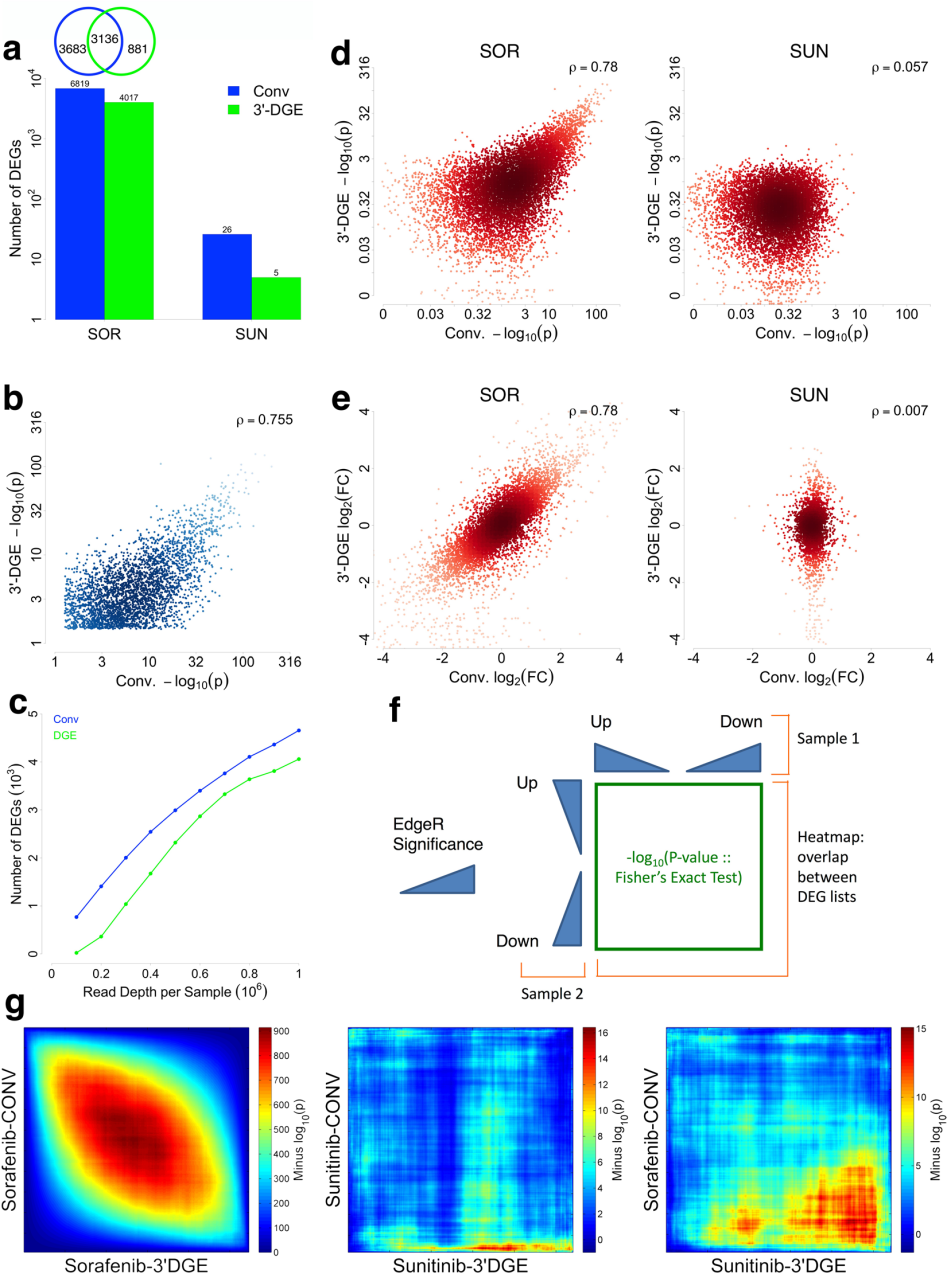
Differential Gene Expression Analysis between Conventional (Conv) and 3′-end Digital Gene Expression (3′-DGE) mRNA Sequencing Methods. (**a**) Control (CTRL) data are compared Sorafenib (SOR) or Sunitinib (SUN) to identify differentially expressed genes (DEGs) using EdgeR for both Conv and 3′-DGE datasets. A gene is defined as differentially expressed using a false discovery rate (FDR) cutoff of 0.1. (**b**) Comparison of statistical significance for the 3,136 shared differentially expressed genes (DEGs) from Sorafenib-treated samples in 3′-DGE and Conv methods with FDR <0.1. The negative base-10 logarithm of the p-value for differential expression is plotted for each technique, with depth of color indicating density of points. Pearson’s correlation coefficient is indicated with the inset text. (**c**) Identification of Differential Expression as a Function of Read Depth. The number of differentially expressed genes (DEGs, FDR < 0.1) was quantified after progressive downsampling of UMI counts from 3′-DGE datasets or read counts from conventional (Conv) datasets (for Sorafenib vs. CTRL). (**d**) Comparison of statistical significance for all genes identified from SOR-treated samples and SUN-treated samples by two methods. The negative base-10 logarithm of the p-value for differential expression is plotted for each technique, with depth of color indicating density of points. The Pearson correlation coefficient is calculated for each treatment. (**e**) Comparison of fold change for all genes identified from SOR-treated samples and SUN-treated samples by two methods. The log base two fold-change is plotted for each technique, with depth of color indicating density of points. The Pearson correlation coefficient is calculated for each treatment. (**f**,**g**) Rank-rank Hypergeometric tests for consistency of differential expression ranking and gene expression signatures. (**f**) All genes for which a p-value for differential expression was calculated were first sorted into up or down regulated genes (as compared to CTRL), and then ranked by statistical significance. The probability of overlap between two different such rank lists was calculated with Fisher’s Exact Test (aka hypergeometric test), and visualized with a heatmap, for all combinations of list cutoffs. (**g**) Pairwise comparisons of SUN- and SOR-treated data for 3′-DGE and conventional. SOR-treated samples show much higher relative statistical significance, as expected, because only SOR induced large changes in gene expression. Note the difference in p-value scales across the three panels, which indicate the relative statistical significance of the results.

The typical endpoint of an mRNA sequencing experiment is testing for statistical significance of differential expression between two or more conditions. There are many software packages available for doing this; here we used EdgeR^26^ (see Methods for specific software settings). Specifically, we compared DMSO vehicle-treated control (CTRL) to either sunitinib (SUN) or sorafenib (SOR) treatment. A typical definition of a “differentially expressed gene” is that which has a false discovery rate (FDR) of lower than 0.1 (Fig. 5a). Both conventional and 3′-DGE data show that sunitinib causes very few genes to be differentially expressed, whereas sorafenib causes differential expression of several thousand genes (Fig. 5a). For the 3,136 sorafenib-associated gene expression changes identified by both conventional and 3′-DGE, there is a strong correlation between the p-values for differential expression (Fig. 5b). However, there are another ~3,600 genes identified as differentially expressed by conventional that are not found by 3′-DGE. The reason for this is a simple statistical power argument due to UMI counting versus read counting. While using UMI counts as the quantification metric in principal improves precision due to removal of PCR bias^15^, at the same time, it reduces effective read depth and therefore statistical power. Prior work, however, has shown that if total read depth is normalized to UMI counts (as opposed to read counts), UMI counts do provide improved statistical power and lower false discovery rate^15^. To address the difference in statistical power with our data, we progressively downsampled counts in the two datasets, and then evaluated the number of DEGs (Fig. 5c). Here, counts refers to UMI counts in the case of 3′-DGE, and read counts in the case of conventional RNASeq We found that one needs ~200,000 more counts per sample with 3′-DGE to achieve a similar number of DEGs. Since on average in our data, ~2-3 read counts gives one UMI count, we estimate that one needs ~500,000 more reads per sample with 3′-DGE to equalize statistical power (as measured by the number of DEGs).

An alternative way of analyzing differential expression is to forgo strict FDR cutoffs and instead analyze trends visible from all 10,121 genes with defined p-values for differential expression. An expected cone-shape that falls along the x = y line is observed as statistical significance varies from weak to strong, consistent with the above hypothesis and further suggestive of concordance between the techniques (Fig. 5d). This concordance is also evident from the strong agreement between the log_2_-fold changes for differential expression from each technique (Fig. 5e). This strongly suggests the biological conclusions drawn from either technique will be concordant. Lastly, we ranked the entire list of 22,811 genes for each mRNAseq technique and drug, and then analyzed their overlap via the rank-rank hypergeometric test^27^ (Fig. 5f,g). Each spot in the heatmap represents the relative statistical significance of overlap between the genes in the ranked list up to that point, starting from the top left corner, via Fisher’s exact test. To distinguish between up and down regulation, we signed the p-value accordingly, placing down-regulated genes with high statistical significance at the bottom of the list (Fig. 5f). As expected, the ranked lists for sorafenib-treated samples from conventional and 3′-DGE show agreement that is more statistically significant than that of the sunitinib-treated samples (indicated by the red color down the diagonal). Likewise, cross comparison between treatments and sequencing type also yield less agreement as expected. We conclude that conventional and 3′-DGE mRNAseq techniques generate highly similar signatures of gene expression for both up and down-regulated genes.

### Comparison of 3′-DGE and Conventional with An Independent Dataset

Can we expect the agreement between 3′-DGE and conventional mRNAseq results to be similar for other datasets? We attempted to answer this question by taking a similar experimental design approach with a different set of RNA, this time from induced pluripotent stem cell (iPSC) lines created from control individuals or those that had a mutation for and clinical presentation of spinal muscular atrophy (SMA), where slightly different random primed library preparation and sequencing approaches were employed (see Methods). SMA is a childhood early onset motor neuron disease^28,29^ where symptoms can be observed as early as 3 months or up to 2-3 years of age. The disease is typically caused by mutations in the SMN1 (Survival Motor Neuron 1) gene, however disease severity varies based on numbers of copies of the related SMN2 gene. As SMN genes control critical RNA biogenesis processes during early development^29^, we anticipate differential gene expression changes even at the iPSC stage without subsequent differentiation. For these analyses, two SMA subject clonal lines and three control lines, with varying numbers of growth replicates, were compared. Growth and disease replicates were combined for comparisons to generate the differentially expressed gene lists conventional mRNAseq and 3′DGEs. While this is not a traditional replicate approach for differential expression analysis, the mixed basis of the samples would only serve to increase sample-to-sample variability, and thus conclusions about consistency of differential expression can be considered as a lower bound that would only improve with other replication designs.

**Figure 6:**
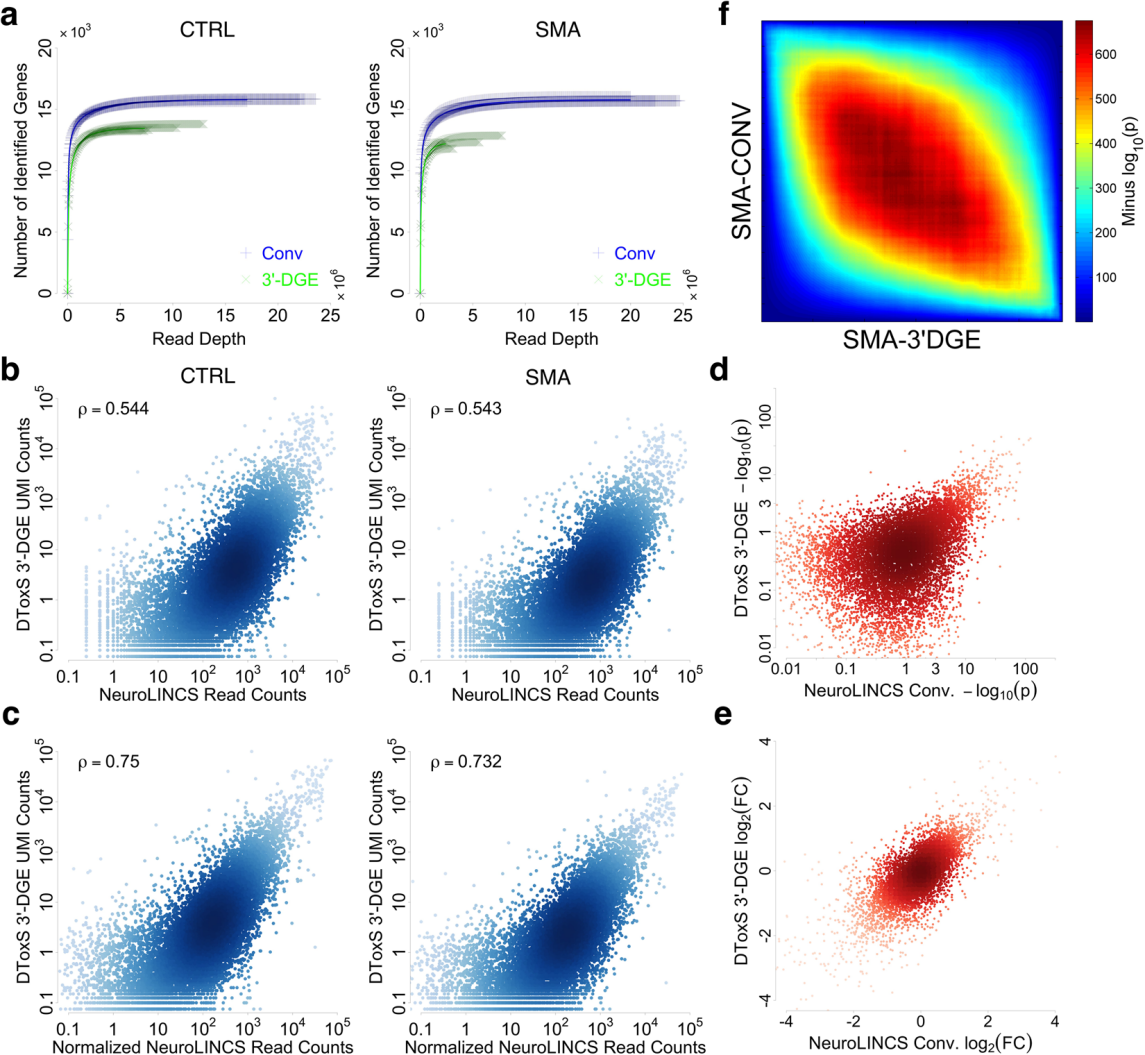
Comparison between Conventional (Conv) and 3′-end Digital Gene Expression (3′-DGE) mRNA Sequencing Methods with an Independent Dataset. (**a**) Sensitivity of gene detection by two methods. Gene-wise reads are removed from every sample in a probability proportional to the abundance of the gene in a sample, to generate a set of the number of identified genes over a range of simulated read depths. The curves for individual replicate samples are shown with the thinner points, showing in general low variability. The average is shown with the solid line. (**b**,**c**) Quantitative gene-wise comparison between two methods. Density of points in scatter plots is indicated by depth of color. Inset text box shows Pearson correlation. In all plots, data are scaled so units are comparable. B. Scatterplots of UMI counts for DToxS’ 3′-DGE versus read counts for NeuroLINCS’ conventional, without normalization by average transcript length. CTRL or SMA refer to the genetic status of the iPS cells (see Methods). (**c**) Scatterplots of UMI counts for DToxS’ 3′-DGE versus transcript length-normalized read counts for NeuroLINCS’ conventional. (**d**,**e**) Comparison of statistical significance (**d**) or fold-change (**e**) for all genes identified from SMA samples by DToxS’ 3′-DGE method and NeuroLINCS’ Conv method. (**d**) The negative base-10 logarithm of the p-value for differential expression is plotted for each technique, with depth of color indicating density of points. **(e)** The log base two fold-change is plotted for each technique, with depth of color indicating density of points. **(f)** Rank-rank Hypergeometric tests for consistency of differential expression ranking and gene expression signatures. All genes for which a p-value for differential expression was calculated were first sorted into up or down regulated genes (as compared to CTRL), and then ranked by statistical significance. The probability of overlap between two different such rank lists was calculated with Fisher’s Exact Test (aka hypergeometric test), and visualized with a heatmap, for all combinations of list cutoffs. Shown here are lists from SMA vs. control for 3′-DGE and conventional.

First, we analyzed sensitivity to detect gene expression as a function of read depth. We observed similar results as before, with conventional having higher sensitivity, detecting ~15% more genes (Fig. 6a), albeit now with both techniques having a higher level of overall detection as compared to the previous dataset. This could be due to different RNA preparation techniques used (TRIzol vs. Qiagen RNAeasy). We observed significant correlation between counts on a gene-by-gene level that again was greatly improved by normalizing conventional data by transcript lengths (Figs 6b,c and S8). Because of the statistical power properties associated with UMI counting discussed above, it is not appropriate to look at strict FDR cutoffs to analyze the number of differentially expressed genes. Alternatively, we analyzed statistical significance (Fig. 6d) and log_2_ fold changes (Fig. 6e) for differential expression and found they were correlated well, and also similarly as before with sorafenib-treated samples. This leads to the resulting ranked gene signatures of differential expression again being highly concordant between the two techniques (Fig. 6f). Thus, we conclude that agreement reported here between 3′-DGE and conventional approaches is likely to be seen across many sample types, as well as across variations of conventional random primed methods.

## Methods

### Cell Culture and RNA Isolation

We used a commercially available cell line to compare the two sequencing methods. Detailed SOPs for culture and RNA isolation are available on our website (www.dtoxs.org) as DToxS SOP CE – 1.0: PromoCell Cardiomyocyte Subculture, DToxS SOP CE – 2.0: PromoCell Cardiomyocyte Plating for Drug Test, DToxS SOP CE – 4.0: Drug Treatment and Cell Lysis, and DToxS SOP A – 1.0: Total RNA Isolation.

Briefly, primary adult human cardiomyocytes (PromoCell, Heidelberg, Germany; Cat: C-12810, Lot: 3042901.2) were subcultured according to manufacturer’s instructions using antibiotic-free myocyte growth medium (PromoCell, Cat: C-22170) supplemented with a mix of 5% fetal calf serum, 0.5 ng/ml EGF, 2 ng/ml FGF, and 5 μg/ml recombinant human insulin (PromoCell, Cat: C-39275). We differentiated fully confluent cells for four weeks under serum starvation and treated cells with DMSO vehicle (Control, CTRL), 2.5 μM sorafenib (SOR), or 0.1 μM sunitinib (SUN) for 48 hours. Total RNA was extracted using TRIzol (Life Technologies, Cat: 15596018). RNA concentration was measured by Qubit 3.0 fluorometric quantitation using the high sensitivity RNA kit (Life Technologies, Cat: Q32852), and RNA quality was assessed by Fragment Analyzer using high sensitivity RNA analysis kit (Advanced Analytical Technologies, Cat: DNF-472). Only the RNA samples that have passed the quality control step with RNA integrity number (RIN) score of 7.0 or above were used. 200 ng of RNA for all samples were then transferred onto a skirted 96-well PCR plate (Eppendorf, Cat: 951020401) at a normalized concentration of 10 ng/μL.

### 3′-DGE Library Preparation

The RNA-seq libraries were prepared according to the Single Cell RNA Barcoding and Sequencing method originally developed for single cell RNA-seq (SCRB-seq; ref.^30^) and adapted to extracted total RNA. Briefly, Poly(A)+ mRNA from extracted total RNA were converted to cDNA decorated with universal adapters, sample-specific barcodes and unique molecular identifiers (UMIs) using a template-switching reverse transcriptase. Decorated cDNA from multiple samples were then pooled, amplified (10 PCR cycles) and prepared for multiplexed sequencing using a modified transposon-based fragmentation approach that enriched for 3′ ends and preserved strand information. A detailed SOP is available at www.dtoxs.org (DToxS SOP A – 6.0: High-throughput mRNA Seq Library Construction for 3′ Digital Gene Expression (DGE)).

### Conventional Random Primed Library Preparation

Conventional sequencing libraries were prepared using 200 ng of total RNA and the TruSeq RNA Library Prep Kit (Illumina, Cat: RS-122-2001) per manufacturer’s instructions, with mRNA was enriched via poly-A-selection using oligoDT beads. RNA was then thermally fragmented and converted to cDNA, adenylated for adaptor ligation and PCR amplified (15 cycles). Prior to sequencing, quality and concentration of the cDNA library was confirmed using Agilent Bioanalyzer and Qubit fluorometric quantitation.

### Sequencing using Illumina HiSeq Platform

Both the random primed and 3′-DGE libraries were sequenced using the Illumina HiSeq. 2500 platform. cDNA libraries were loaded onto Illumina flowcells using the Illumina c-Bot, and conventional libraries were sequenced with 100 nucleotide paired-end reads per manufacturer’s instruction, whereas 3′-DGE used a custom paired end protocol with 26 bp on the first read, and 46 on the second. Detailed SOPs for the 3′-DGE sequencing are available on www.dtoxs.org as described above.

### iPSC Cell Culture, RNA Isolation and Conventional Sequencing with iPSC Data

Human iPSCs were generated using episomal reprogramming and validated using quality control methods, including Pluritest, G-band karyotype analysis and Short Tandem Repeat identity test assays as described previously^29^. iPSCs were maintained in 6-well tissue culture plates coated with Corning Growth Factor Reduced Matrigel Matrix (Cat # 354230). iPSCs were passaged every 7 days or when the cultures reached 80–90% confluency using the StemPro EZPassaging Tool (Life Technologies, Cat # 23181010). To prepare cells for cell pellets, each iPSC line was seeded into two 6-well plates at a 1:6 dilution and allowed to reach ~80% confluency (approximately 1.5 to 2 ×10^6^ cells per well). On the day of collection, any visible spontaneous differentiation was removed using a pipette tip. The spent media was then aspirated and the cells were rinsed with chilled PBS. 1 ml of chilled PBS was added to each well and the cells were lifted gently from the plate using a Corning cell scraper (Cat # CC3010). Two wells from each plate were pooled together into a chilled 15 ml conical and labeled as replicate #1. This was repeated until three replicates were collected per sample. Each replicate was then evenly distributed into 5 chilled 0.5 ml Eppendorf tubes. The Eppendorf tubes were labeled with the cell line name, passage number, collection date and replicate number. Cells were then pelleted by centrifuging each sample for 5 minutes at 1000 rpm. The PBS was aspirated and the cell pellets were then flash frozen by briefly submerging each tube in liquid nitrogen. The samples were sent for RNA preps and sequencing. Full details relating to Control and SMA cell lines are also available at http://lincsportal.ccs.miami.edu/dcic-portal/.

Total RNA were isolated using the Qiagen RNeasy Kit and QIAshredder. RNA QC were analyzed on the Agilent 2100 Bioanalyzer which indicated that all RIN values were 10. RNA-Seq libraries were made with 1 ug of RNA using the Illumina non-stranded TruSeq mRNA v2 protocol. Libraries were quantified using the KAPA library quant kit and sequenced on the HiSeq. 2500 using 100 cycles across three lanes to obtain paired-end reads 100 base pairs in length.

### Defining and Alignment to Reference Genome

For the conventional method, we obtained the RefSeq genome sequence file (hg19) from the UCSC website (http://hgdownload.cse.ucsc.edu/downloads.html#human). We created the gene annotation file by downloading a list of RefSeq genes from *Table Browser* (http://genome.ucsc.edu/cgi-bin/hgTables) and then converting it to the GTF format using *genePredToGtf* (http://hgdownload.cse.ucsc.edu/admin/exe). For 3′-DGE, we mapped the reads using BWA on the mRNA RefSeq FASTA files downloaded from UCSC hg19 (RefSeq) for which we trimmed the homopolymers of A occasionally present at the end of the full mRNA transcripts. There are a common set of 22,081 genes which we used as a basis of comparison between the two techniques throughout the manuscript (Table S1). We used STAR (Dobin *et al*. 2013) with the default parameter settings to align the conventional mRNAseq data (PromoCell cardiomyocytes) to the reference described above, and then counted the number of the sequence fragments uniquely aligned to each gene by the *featureCounts* program from a sequence alignment suite *Subread* using the UCSC reference gene annotation. For 3′-DGE data, a custom python script is used (available upon request and from GEO). First, reads are aligned using BWA, and counts of specifically or non-specifically aligned reads (i.e., aligning to one or more than one gene with high confidence) are calculated. Next, the number of distinct unique molecular identifier (UMI) sequences embedded in those aligned reads are counted, giving the UMI counts.

We compared alignment of 3′-DGE data using both annotations, and found good correlation on the level of read counts (Fig. S10). There are two classes of differences we observed. First, there are a group of 67 genes that are highly expressed in both, but are consistently more readily aligned to using the mRNA RefSeq annotation (new Supplementary Table S8). These genes cause on average ~500,000 more read counts per sample (Fig. S11). Second, there are a group of 32 genes that are lowly to moderately expressed, but are not readily aligned to in the mRNA RefSeq annotation (see Supplementary Table S9). Both sets of genes should be interpreted with caution when using the 3′-DGE approach.

### Computational Downsampling of Sequencing Depth

To compare datasets on an equivalent sequencing depth basis, we computationally removed read counts with an iterative algorithm (Figs S4,S5). First, all genes with very low expression are removed (<4 read counts). Then, a particular gene is randomly selected with probability proportional to its count representation amongst all genes, and a read is removed from this gene. We removed UMI counts from the selected gene probabilistically, according to the ratio of UMI counts to read counts (always less than one, and a gene-specific estimate of PCR bias). The process is repeated using probabilities estimated at the first iteration until all read counts are removed. We performed the process 16 times for each sample to ensure the stochastic nature of removal did not affect our results (Fig. S6).

### Differential power to identify specific biological processes

To determine the ability of the different mRNAseq methods to identify particular biological processes in the GO biological process ontology, we conducted a power analysis. We assumed the enrichment analysis was conducted using a Fisher’s exact test and the comprehensive GO Biological Processes ontology (5192 processes). The power calculation function for the Fisher’s exact test in the statmod R package was used for this purpose. A significance criterion of 0.05 was used. We focused on two cases. First, we specifically focused on genes uniquely associated with the UCSC (4270 genes) and the Broad reference (1084 genes). For both reference gene lists we determined the processes that were associated with a power > 0.8 while the remaining reference gene list had a power < 0.8 (Table S7). It should be noted that this approach can be considered conservative, because the genes that were shared between the two references were not taken into account in this analysis. Second, we specifically focused on genes that were detected across the cardiomyocyte data for each technique. For each individual sample that was sequenced using both methods, we determined the number of genes for which a count > 0 was obtained, i.e. that could be detected using both the conventional or 3′-DGE method. After calculating the power to detect a biological process we calculated the mean power to detect each of the biological processes in the GO Biological Processes database, for each of the two methods, looking for power loss (Fig. S9).

### Data Normalization

For conventional data, we divided read counts by the average transcript length. These lengths were obtained from http://genome.ucsc.edu/cgi-bin/hgTables, by summing the length of all exons in a transcript into a transcript length, and then averaging this transcript length across all transcripts of each gene.

### Differential Expression Analysis

We performed differential expression analysis for the cardiomyocyte data with edgeR^26^ starting with tables of counts for any technique. Differential expression analysis consists of the following steps within edgeR: normalization by trimmed mean of M-values^31^, empirical Bayes estimation of sample dispersion^32^, and exact test for negative-binomial sample comparison^33^. A detailed standard operating procedure (SOP) document is available at www.dtoxs.org, and all code is available upon request.

### Rank-rank hypergeometric test

The significance of the overlap between two lists of differentially expressed genes (DEGs) was calculated using the rank-rank hypergeometric test^27^. First, for all genes, we calculated the negative log_10_ of the p-value for differential expression (−log_10_*p*), and multiplied by negative one if a gene was downregulated. Genes were ranked by signed −log_10_*p*, placing the most significantly up-regulated genes at the top and the most significantly down-regulated genes at the bottom of the list. The number of overlapping genes of the top *x* genes of one list and the top *y* genes of the other was counted at every 10^th^ *x-y* combination, and Fisher’s exact test was used to calculate significance of the overlap. If the overlap was greater than expected the right-tailed Fisher’s Exact test was used, otherwise the left-tailed Fisher’s exact test was used. The resulting heatmap of p-values from Fisher’s Exact text was visualized with the MATLAB function imagesc.

### Data Access

All the read counts datasets used in this study can be accessed at the DToxS website (https://martip03.u.hpc.mssm.edu/xiong_paper/mRNAseq-Counts-Datasets.R20170117.html), and are also deposited on GEO (GSE98432—see README text files in the SubSeries pages for detailed information). In the Conv group of the NeuroLINCS-DGE-Conv datasets, the read counts table is adapted from the level-3 normalized dataset (Dataset ID: LDS-1355, http://lincsportal.ccs.miami.edu/datasets/#/view/LDS-1356), by extracting the read counts of a common set of genes shared between the datasets from both sequencing methods.

## Electronic supplementary material

Supplemental Figures

Supplementary Dataset S1-S9
