# Supplementary material for: A Comparison of mRNA Sequencing with Random Primed and 3′-Directed Libraries
Source: Sci Rep. 2017 Nov 7;7:14626. doi: 10.1038/s41598-017-14892-x (PMC5676863; doi:10.1038/s41598-017-14892-x)
Supplement: Supplementary file 1 — Supplemental Figures [file 41598_2017_14892_MOESM1_ESM.pdf]

A Comparison of mRNA Sequencing with Random Primed and 3'-Directed Libraries

Yuguang Xiong[1], Magali Soumillon[2,10], Jie Wu[3,4], Jens Hansen[1], Bin Hu[1], Johan G.C. van Hasselt[1], Gomathi Jayaraman[1], Ryan Lim[3,4], Mehdi Bouhaddou[1], Loren Ornelas[5,6], Jim Bochicchio[2], Lindsay Lenaeus[5,6], Jennifer Stocksdale[4], Jaehee Shim[1], Emilda Gomez[5,6], Dhruv Sareen[5,6,7], Clive Svendsen[5,6,7], Leslie M. Thompson[3,4,8], Milind Mahajan[9], Ravi Iyengar[1], Eric A. Sobie[1], Evren U. Azeloglu[1,#], Marc R. Birtwistle[1,11,#]

[1]Department of Pharmacological Sciences and DToxS LINCS Center, Icahn School of Medicine at Mount Sinai, New York, NY USA

[2]Broad Institute of MIT and Harvard, Cambridge, MA USA

[3]Department of Biological Chemistry, University of California, Irvine, CA USA

[4]UCI MIND, University of California, Irvine, CA USA

[5]Board of Governors-Regenerative Medicine Institute, Cedars-Sinai Medical Center, Los Angeles, CA, USA

[6]iPSC Core, The David and Janet Polak Foundation Stem Cell Core Laboratory, Los Angeles, CA, USA

[7]Department of Biomedical Sciences, Cedars-Sinai Medical Center, Los Angeles, CA, USA

[8]Department of Psychiatry and Human Behavior, Neurobiology and Behavior, University of California, Irvine, CA USA

[9]Department of Genetics, Icahn School of Medicine at Mount Sinai, New York, NY

[10]Present Address: Berkeley Lights, Inc. 5858 Horton St., Emeryville, CA 94608

[11]Present Address: Department of Chemical and Biomolecular Engineering, Clemson University, SC USA

[#]To whom all correspondence should be addressed: evren.azeloglu@mssm.edu or marc.birtwistle@mssm.edu

Xiong, et al. Figure S1

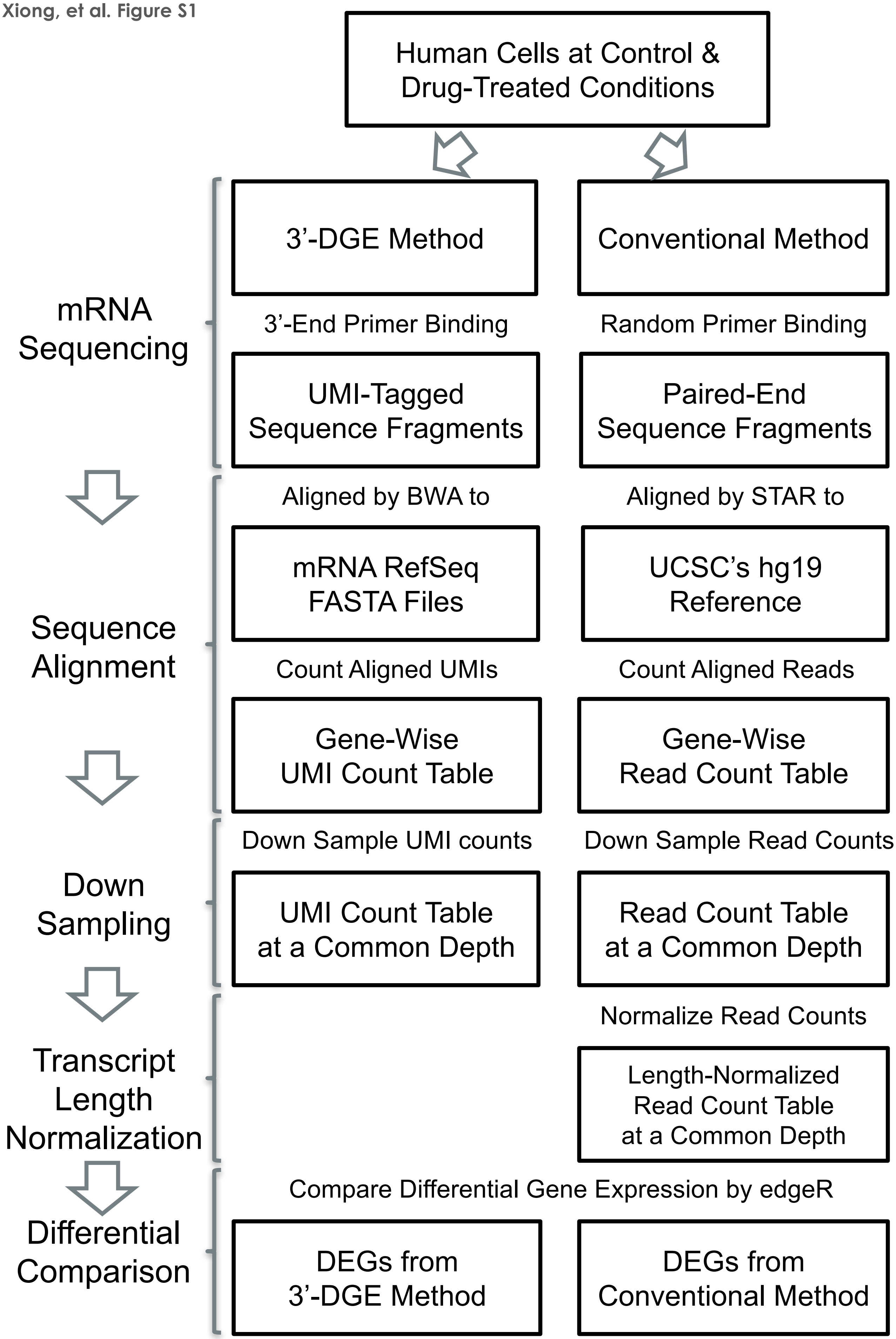

Figure S1. The Workflow of Comparing Two mRNA Sequencing Methods: the 3'-end Digital Gene Expression (3'-DGE) Method and the Conventional Random Primer-binding Method.

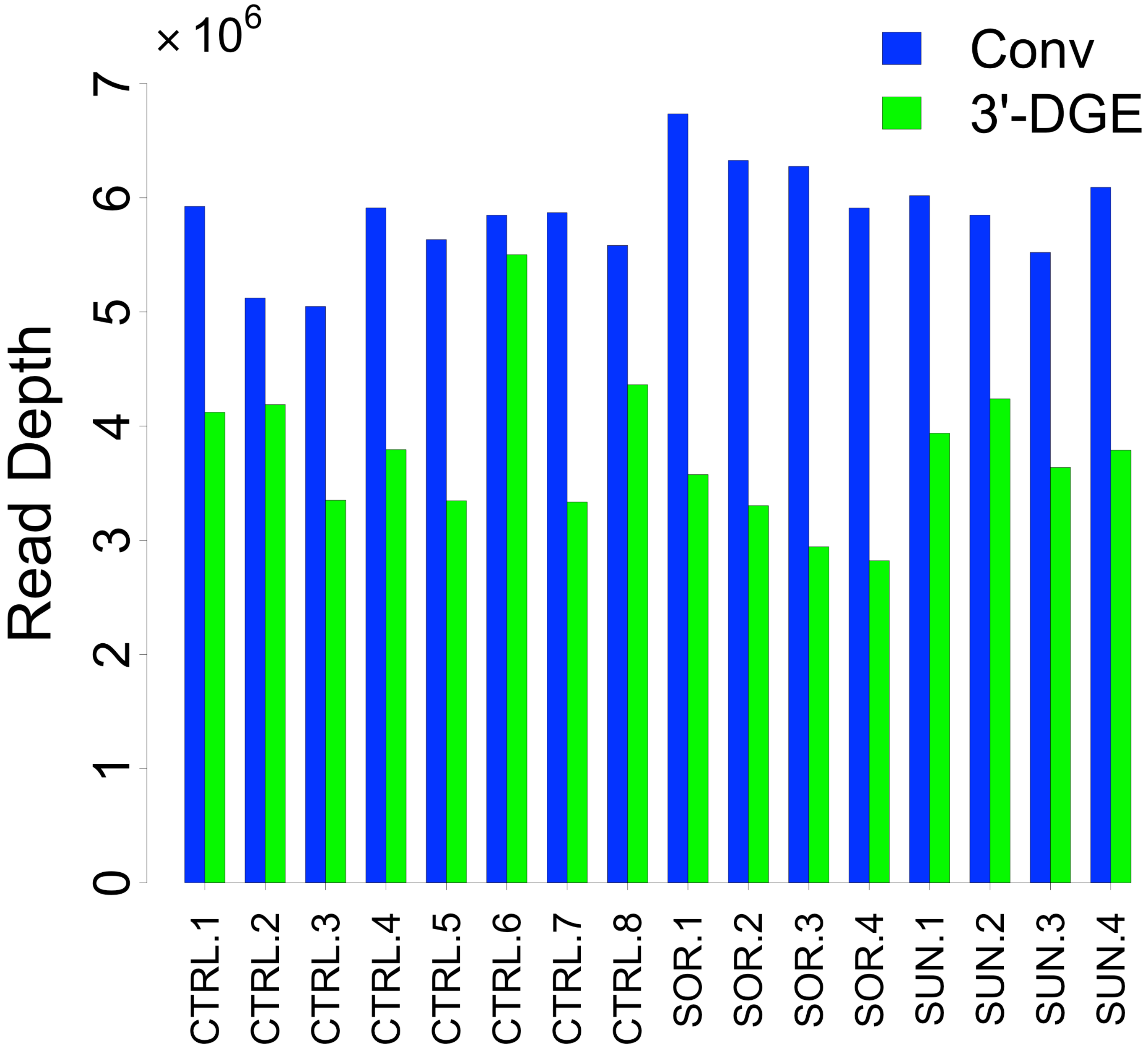

Figure S2. Read Depth Across Multiple Samples for Conventional (Conv) and 3'-end Digital Gene Expression (3'-DGE) Methods. The total number of uniquely aligned reads is plotted for each sample across the three treatment conditions: control (DMSO-CTRL), Sorafenib (SOR), and Sunitinib (SUN)). All samples show consistent read depth.

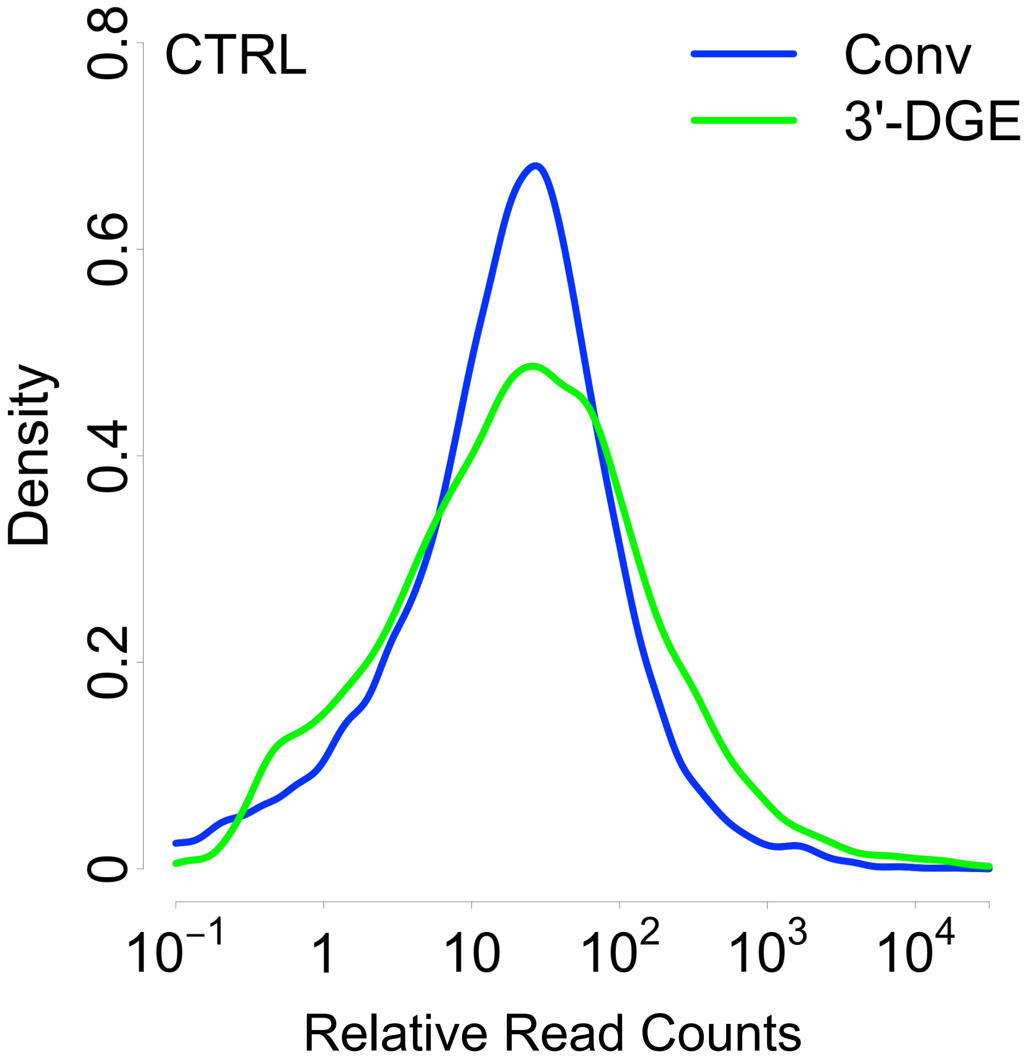

Figure S3. Relative Read Count Distributions for Conventional and 3'-DGE Methods. The mean read counts for each gene across the eight control samples, downsampled to a common read depth (2.8 million per sample) was calculated, and for conventional, this value was divided by transcript length. The probability density was estimated by the *density* function of R package *stats*.

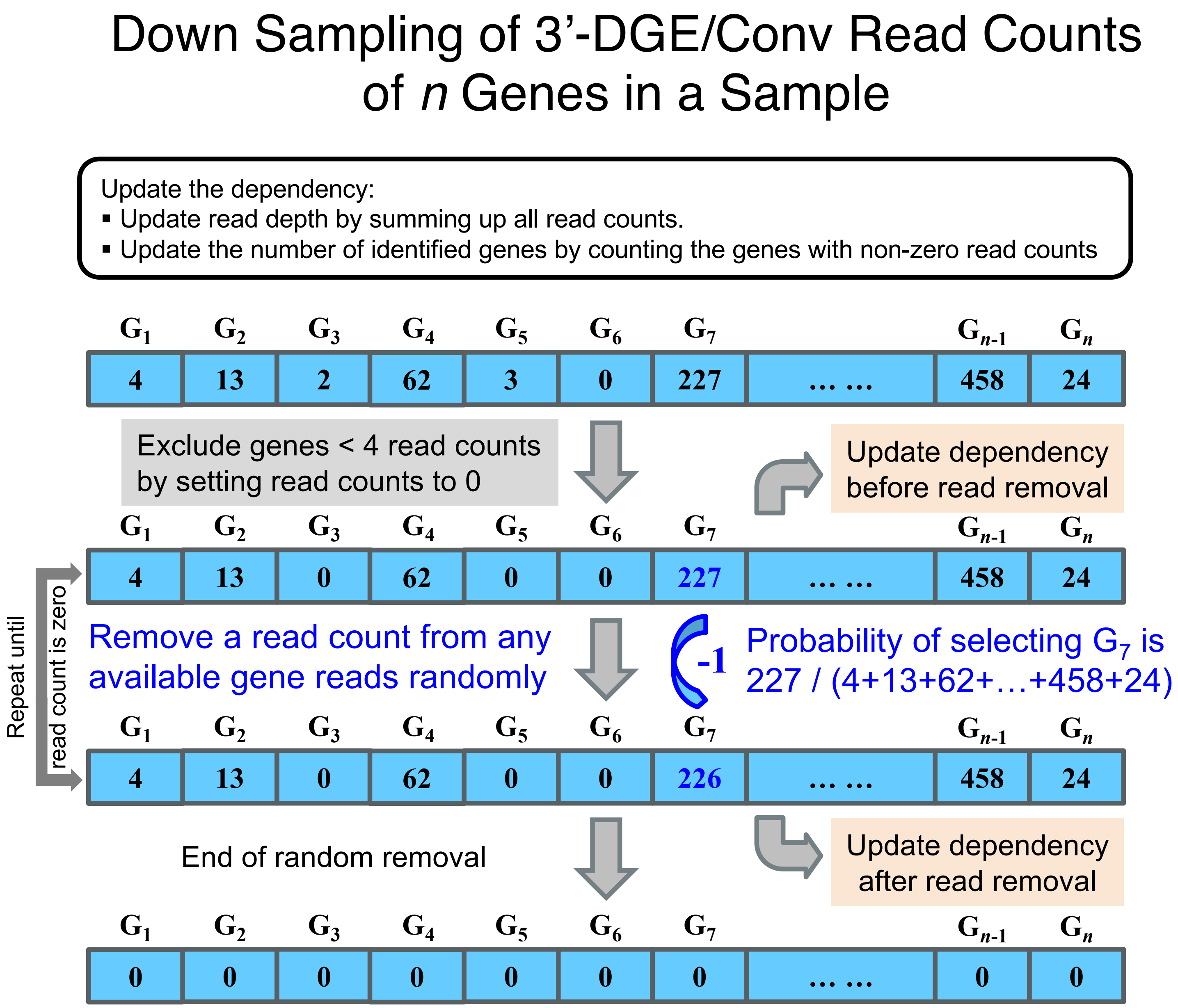

Figure S4. Down-sampling Read Counts in 3'-end Digital Gene Expression (3'-DGE) and Conventional (Conv) Sequencing Methods.

Down Sampling of 3'-DGE Read Counts & UMI Counts of *n* Genes in a Sample

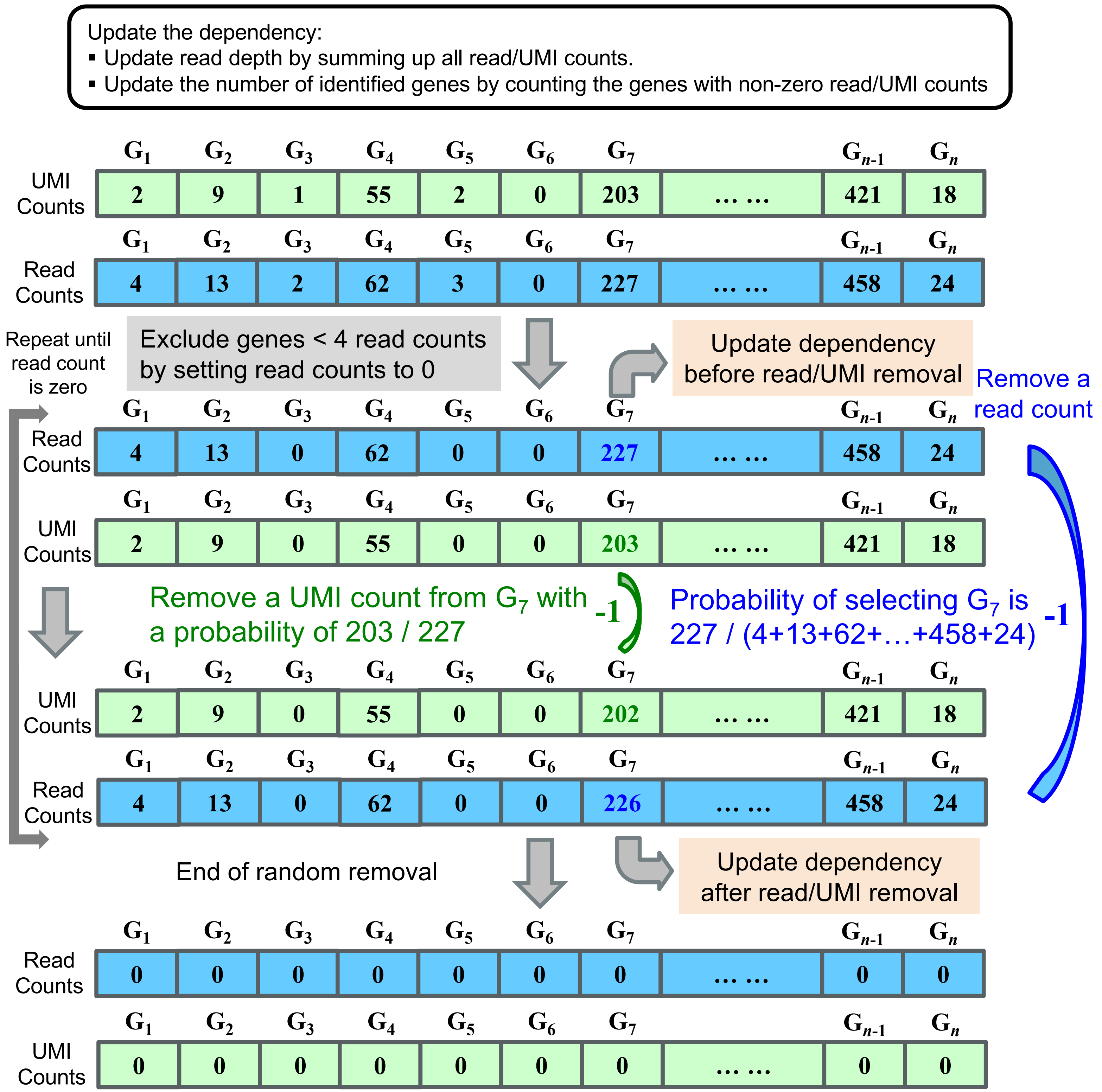

Figure S5. Down-sampling Read Counts and Unique Molecular Identifier Counts for the 3'-Digital Gene Expression (3'-DGE) Method.

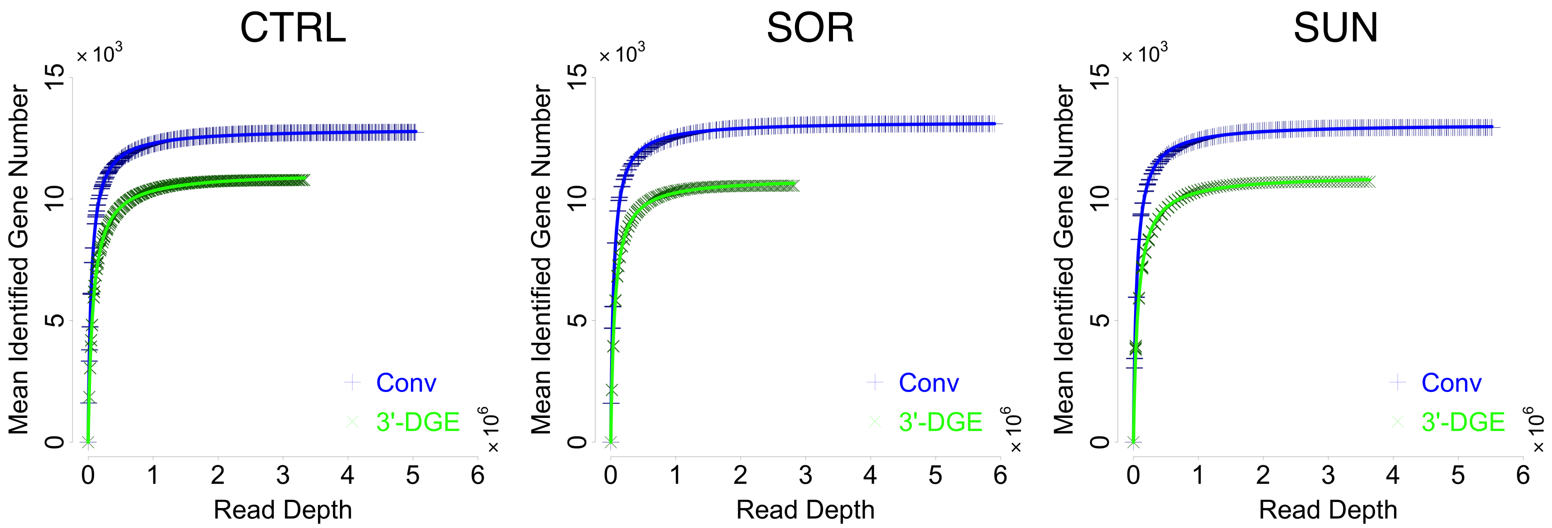

Figure S6. Variability in the Read Removal Process. Random read removal was performed 16 independent times, and the range of variability across those runs is not visible on this chart despite 16 different runs being plotted, indicating a highly reproducible simulation algorithm for read removal on the level of identified genes.

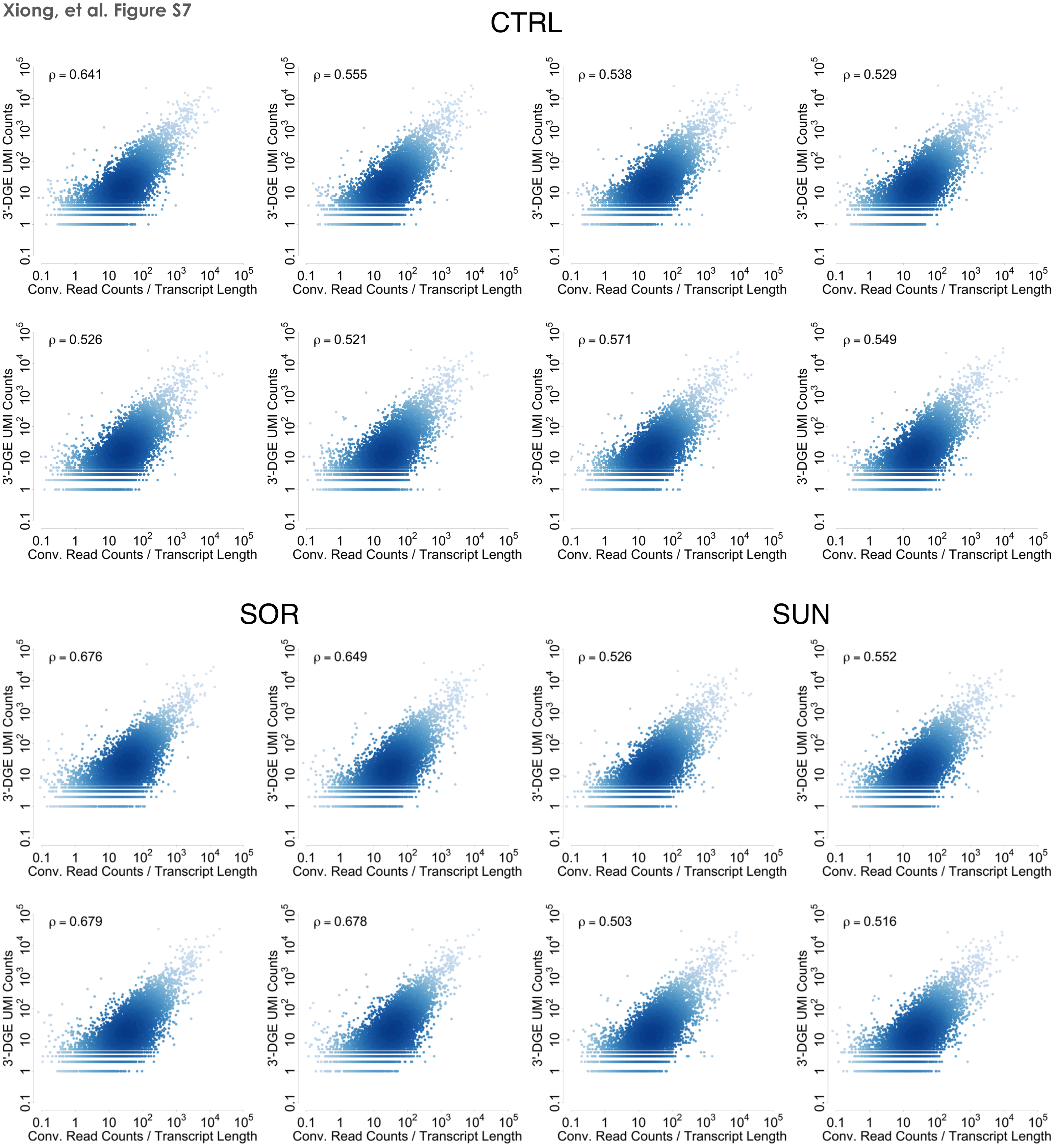

Figure S7. Sample-to-Sample Comparison between Two Techniques. Datasets are down-sampled to a common read depth of 2.8 million reads, and then gene-by-gene comparisons are made via scatter plots. To generate a reduced UMI count dataset, upon removal of a read count, UMI counts were removed with probability proportional to the ratio between UMI counts and read counts for that gene (accounting for PCR bias). Density of points in scatter plots is indicated by depth of color. Inset text box shows Pearson correlation. In all plots, data are scaled so units are comparable. There are eight CTRL samples, four SOR samples, and four SUN samples. All are biological replicates.

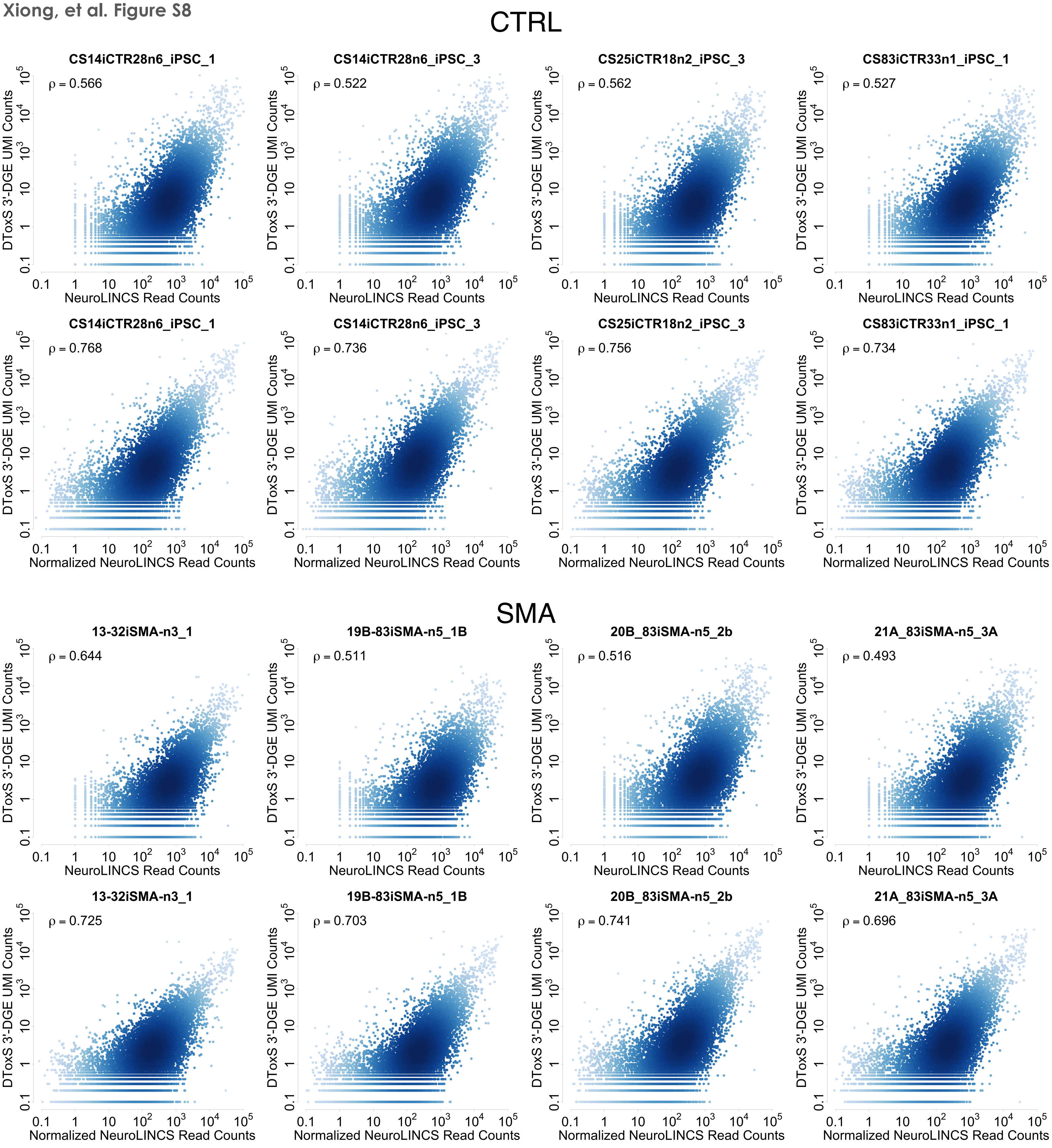

Figure S8. Sample-to-Sample Comparison between 3'-end Digital Gene Expression (3'-DGE) and Independent Conventional Techniques. Density of points in scatter plots is indicated by depth of color. Inset text box shows Pearson correlation. In all plots, data are scaled so units are comparable. There are four CTRL samples and four SMA samples. All are biological replicates.

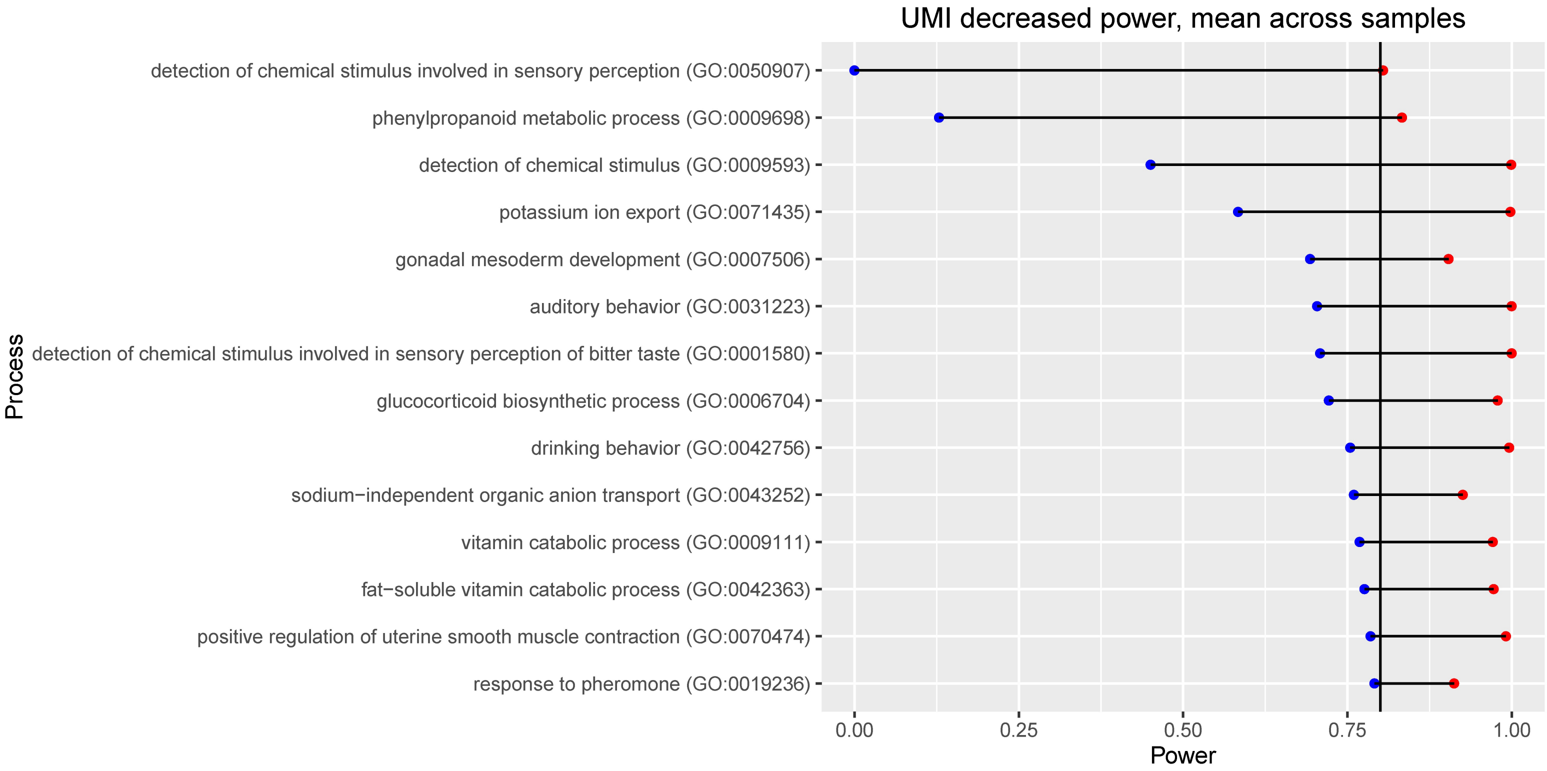

Figure S9. Power of 3’-DGE and Conventional to Detect Biological Processes Based on Genes Detected in the PromoCell Datasets. Mean statistical power across available samples to detect a biological process present in the GO Biological Process ontology using the 3’-DGE method (blue circles) and the conventional mRNA sequencing method (red circles). Shown are only biological processes where the mean power for the 3’-DGE method is <0.80 while the conventional method has a power >0.80 at a significance criterion of 0.05.

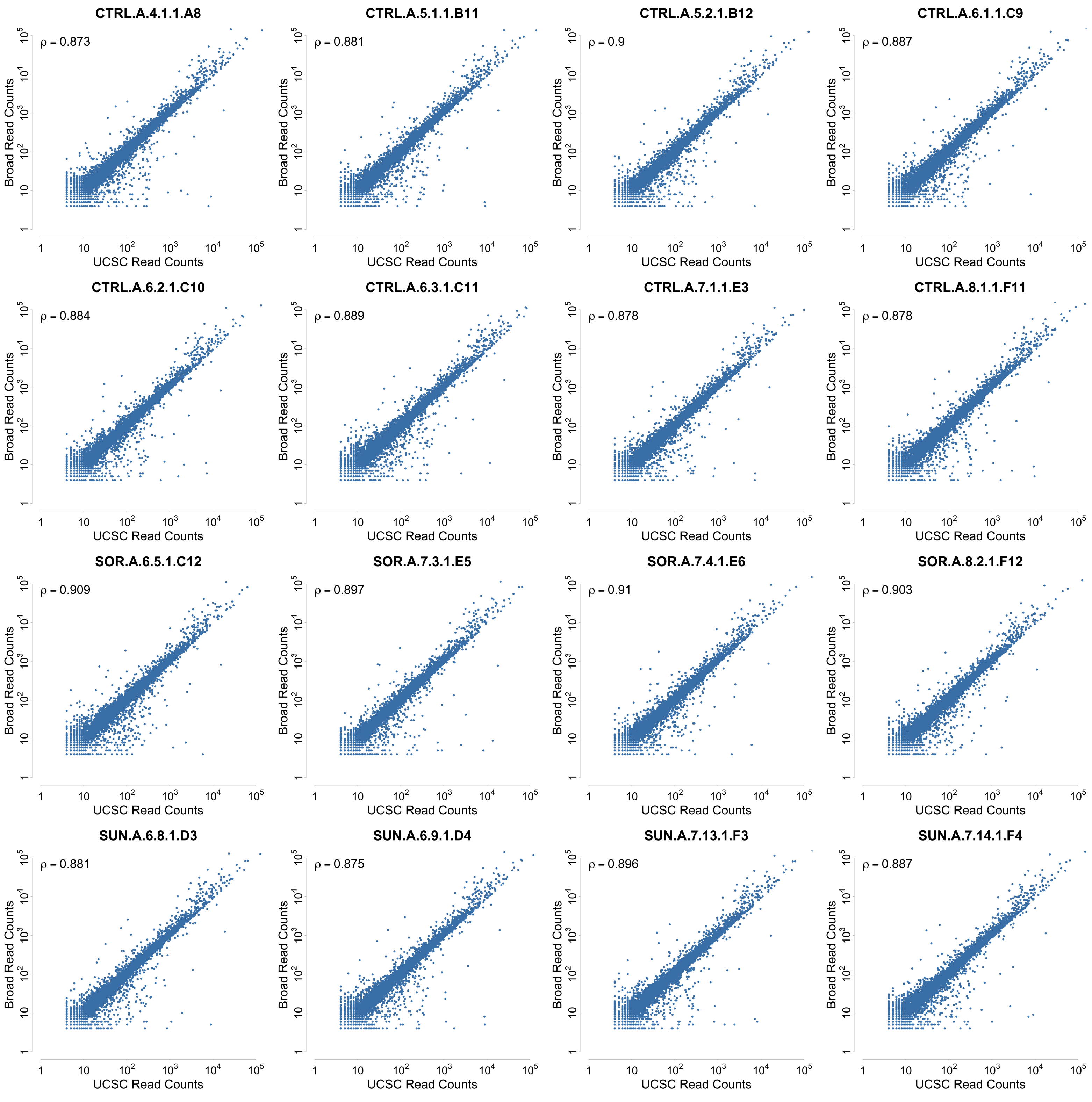

Figure S10. Comparison of read counts for 3'-DGE dataset after alignment to the UCSC RefSeq annotation used for conventional dataset, and to the mRNA RefSeq annotation provided by the Broad.

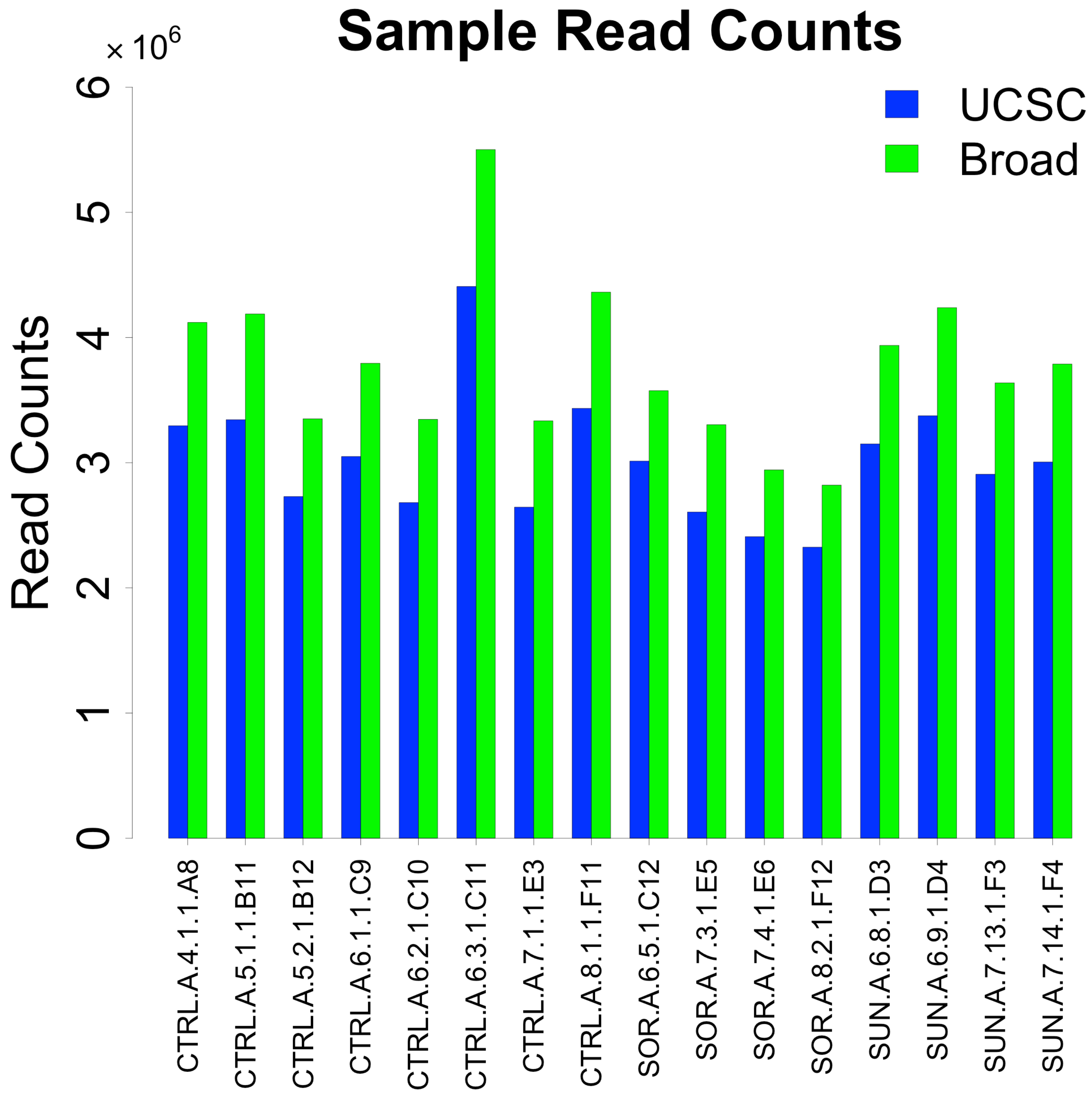

Figure S11. Sample-level read counts of 3'-DGE dataset aligned to the UCSC RefSeq (hg19) annotation versus the read counts when aligned to the mRNA RefSeq FASTA provided by the Broad. Quantitatively, the results are very similar and uniform across samples, with the Broad-provided reference generally providing more aligned reads. These increased read counts are primarily attributable to a group of 67 genes (Table S8). See Methods for details of differences.
